# Life-Cycle-Based Multicriteria Sustainability Evaluation of Industrial Parks: A Case Study in China

**DOI:** 10.1100/2012/917830

**Published:** 2012-12-05

**Authors:** Jin Yang, Bin Chen, Jing Qi, Shiyi Zhou, Meiming Jiang

**Affiliations:** ^1^State Key Joint Laboratory of Environmental Simulation and Pollution Control, School of Environment, Beijing Normal University, Beijing 100875, China; ^2^Beijing Development Area Co. Ltd, Beijing 100176, China

## Abstract

Along with increasing concerns on environmental protection and global warming mitigation, new industrial organization modes such as “Ecoindustrial Park” and “Low Carbon Industrial Park” are emerging. Since ecoindustrial parks and low carbon industrial parks may offer multifaceted benefits to the users, it naturally follows that the sustainability assessment of the industrial parks ought to adopt a multicriteria methodology. In this paper, a multicriteria sustainable evaluation framework is proposed in combination with the life cycle analysis and applied to a low carbon and high end industrial park (LCHE) in Beijing, China. Results show that the LCHE industrial park can contribute to both energy-saving and greenhouse gas emission mitigations compared with other industrial parks. In terms of economic performance, although the economic profits are considerable, the investment per constructed area is relatively high. The results of sustainable analysis of the LCHE industrial park can thus shed light on future upgrading of industrial parks.

## 1. Introduction

Industrial parks are defined as the land areas developed and subdivided into plots according to their integrated plans with provisions for roads, transport, and public utilities for the use of a group of industrialists [1]. Industrial parks usually function as small cities with complete infrastructural facilities, intermaterial and information flows, and semi-artificial environmental conditions. They assimilate materials from the outside and deliver products to the human society. As a cardinal unit of economic development, industrial parks have been playing an important role in the national development strategies of many countries and have been irreplaceable where economic development is concerned [2]. There are several interchangeable terms for industrial parks, which often vary depending on the scope and type of operations, for example, business parks, office parks, science and research parks, hi-tech centers, and bio-technology parks [3]. As many industrial types exist, it opens up an opportunity to establish the most sustainable or ecoefficient industrial park.

Considering the coordination of economic development and environmental protection, the concept of “Eco-industrial Park” (EIP) was proposed and defined as “a community of manufacturing and service businesses located together on a common property.” Member businesses seek enhanced environmental, economic, and social performance through collaboration in managing environmental and resource issues. By working together, the community of businesses seeks a collective benefit that is greater than the sum of individual benefits each company would realize by only optimizing its individual performance [4–7]. In China, accelerated by the “National Pilot EIP Program” and “National Pilot Circular Economy Zone Program,” 60 industrial parks have received approval to be developed into national pilot EIPs [8].

The world is experiencing a behavior transition triggered by climate change in recent years. To probe into the status and trend of global warming, research has been widely conducted focusing on complex social-economic systems [9–15]. With the concurrent concerns on climate change, the construction of low carbon industrial parks is emphasized to favor the development of low carbon economy in China. Low carbon industrial park is an updated EIP with carbon emission control taken into consideration. The establishment of low carbon industrial park aims at minimizing the carbon emissions and environmental impacts while maximizing the economic output. It involves low carbon building, low carbon lifestyle, preferable environment, and high economic efficiency. Now, some pilot low carbon industrial parks have already been established in China, for example, the Shanghai Expo Area.

Although the construction of EIPs or low carbon industrial parks is quite popular, the sustainability assessment of industrial parks is a topic that has not been well documented. It appears that most sustainability studies on industrial parks fail to link the infrastructure developments to the energy system thus focusing more on limited aspects such as direct energy consumption [16], environmental impacts [17], economic or social performance [18], or inner metabolism [19].

Since EIP and low carbon industrial park may offer multifaceted benefits to the users, it naturally follows that any sustainability assessment of the industrial park ought to adopt a multi criteria methodology. Hence, sustainability assessment of industrial parks should be illustrated considering the dimensions of environmental, economic, resource sustainability. Based on the concept of life cycle analysis (LCA), which is commonly used to trace the energy consumption and carbon emissions of artificial ecosystems [20–25], especially buildings [26–30], this paper aims to propose a sustainability evaluation framework which integrates the environmental impacts, economic output, and resource depletion and apply it to a low-carbon and high-end (LCHE) industrial park in China. The remainder is organized as follows: Section 2 describes the evaluation framework, that is, the environmental, economic, and resource depletion evaluation indicators. In Section 3, the case concerned is introduced. The accounting results are integrated and demonstrated in Section 4. Finally, Section 5 presents the conclusions of this paper.

## 2. Methodology

The general requirements for selection of impact criteria are reliability, measurability, and relevance/usefulness [31]. Based on these disciplines and the LCA framework, an outline is proposed to evaluate the sustainability (including environmental, economic, and resource performance) of the industrial parks. After clarifying the objective and system boundary, an inventory analysis is performed firstly in terms of the total material inputs. Then, the calculated environmental emission, economic investment, and resource consumption are demonstrated, based on which a set of indicators are finally calculated. Thus, a LCA based multi criteria sustainability evaluation framework is established. The proposed indicators are listed in Table 1.

### 2.1. Environmental Evaluation

As climate change is attracting more and more public concerns, greenhouse gas emission is selected to represent the environmental impact of industrial parks in this sustainability evaluation framework. Both carbon sources and sinks are considered in the construction stage using indicators of carbon emission density and the greening rate, which can be viewed as the pillars of an environmental managerial decision. Specifically, greenhouse gas emission intensity, which is a measurement of environmental impact in the operation phase, entails the environmental cost of economic development. The higher the greenhouse gas emission intensity, the larger pressure exerted on climate change and environmental mitigation. In the dismantling phase, greenhouse gas emission removal rate is used to quantify the role of material recycling in mitigating greenhouse gas emission.

### 2.2. Economic Evaluation

The economic sustainability dimension includes aspects directly and indirectly quantifiable in monetary terms such as, respectively, total investment per area, and economic output per area, which are reflections of costs and economic vigor of industrial parks [31]. The computations of economic indicators are also based on the life cycle concept, including economic inputs per constructed area on building works and infrastructure installation and the economic output per area in the operation stage of industrial parks.

### 2.3. Resource Depletion

The resource depletion in the construction phase is illustrated by two indicators, that is, energy density and water recycling rate. Energy density is defined as the embodied energy consumption per construction area. It is a criterion for energy performance comparison among different industrial parks. As waste water is treated and reused through a waste water treatment system in most industrial parks, the water recycling rate is employed to measure the renewability of industrial parks. In the operation stage, Energy intensity, which is specified as the energy consumption per $, is employed to demonstrate the energy cost along with economic development. It also functions as a goal-function for tradeoffs between energy saving and economic development. Energy saved by material recycling in the dismantling stage is also signified by the indicator of energy reduction rate.

## 3. Case Study

### 3.1. Description of Study Site

The concerned LCHE industrial park is located in the northeast of Beijing, China. There is a convenient transportation network with Jinghu national highway, light rail Yizhuang line, the Fifth Ring Road, and other thoroughfares connected to this area. In this industrial park, totally 159 enterprises, covering high-end economies such as intelligent, innovative, and design ones, are concentrated. Capital investments of settled enterprises in this industrial park amount to $82.53 billion, and more than 5000 employments are provided.

**Figure 1:**
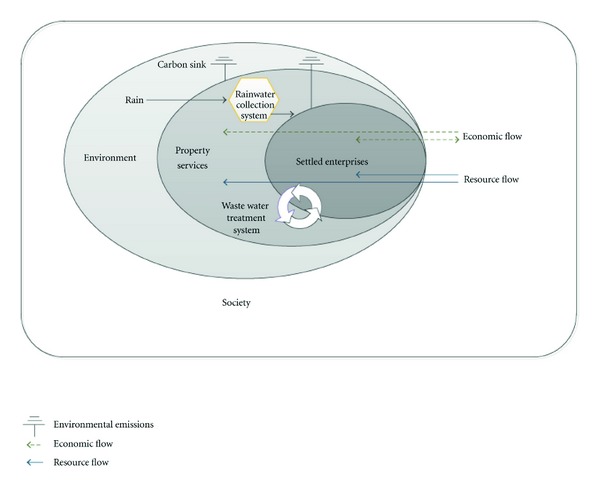
Components of the LCHE industrial park.

The industrial park has a floor space of 0.174 million square meters and built up area of 0.336 million square meters. It is divided into Zone A and Zone B. Zone B with 43 office buildings (3-4 floor building) is studied in this paper. As shown in Figure 1, the concerned Zone B consists of settled enterprises, property services, and the supporting environment of the industrial parks. A rainwater collection system and a waste water treatment system are installed as auxiliary engineering. The plot ratio of buildings is 0.77 and the building intensity is 31.62%.

### 3.2. Goal and Scope

The study presented in this paper uses an LCA framework as a tool to conduct the sustainability evaluation of a LCHE industrial park in Beijing, China. An inventory of the materials involved in the construction, operation, and dismantling stages is used to calculate the environmental, economic, and resource conditions. The goal of the study is to investigate the economic performance, environmental consequences, and resource depletion of the industrial park and identify if the so-called low carbon industry is more sustainable than other industrial estates.

### 3.3. System Boundary

**Figure 2:**
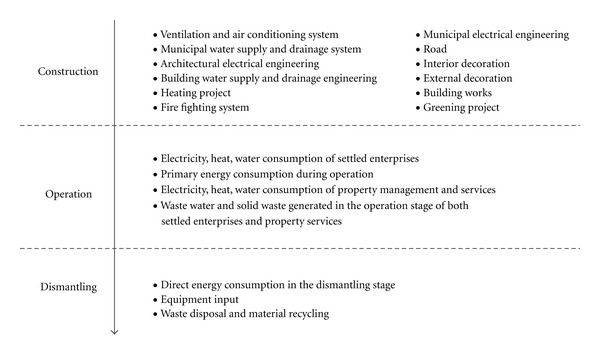
System boundary of sustainability evaluation.

All material and facility inputs in the lifetime (construction, operation, and dismantling phases) of the industrial park are accounted. In the construction phase, 12 subsystems are accounted including ventilation and air conditioning system, municipal water supply and drainage system, architectural electrical engineering, building water supply and drainage engineering, heating project, firefighting system, municipal electrical engineering, interior decoration, road, external decoration, building works, and greening project. The operation of the industrial parks included in the system boundary is based on electricity, heat, and water consumption. In the dismantling phase, material inputs of both disposal and recycling are combined. The system boundary of the evaluation is depicted in Figure 2.

The analysis took into account the entire life cycle of this industrial park. The construction of this industrial park takes 20 months while the operation period is expected to be 47 years and 4 months.

### 3.4. Data Sources

Data for the LCHE industrial park is kindly provided by the developer, Beijing Economic-Technological Development Area. The data include the price and quantity of construction materials in construction as well as operation phases, and environmental inputs. The raw data are then converted to economic, greenhouse gas emission, and embodied energy flows. The greenhouse gas emission and embodied energy coefficients are derived from LCB [33] and LCE [34].

## 4. Results and Discussions

### 4.1. Environmental Emissions

**Figure 3:**
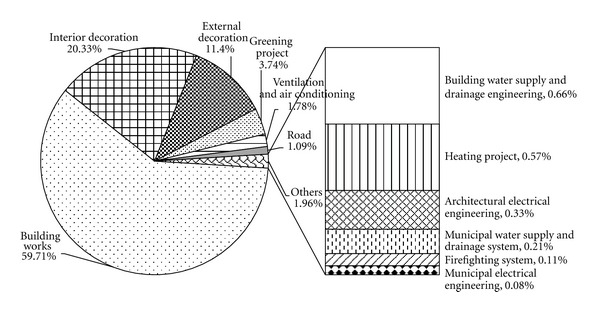
Greenhouse gas emission sources of the construction phase.

**Figure 4:**
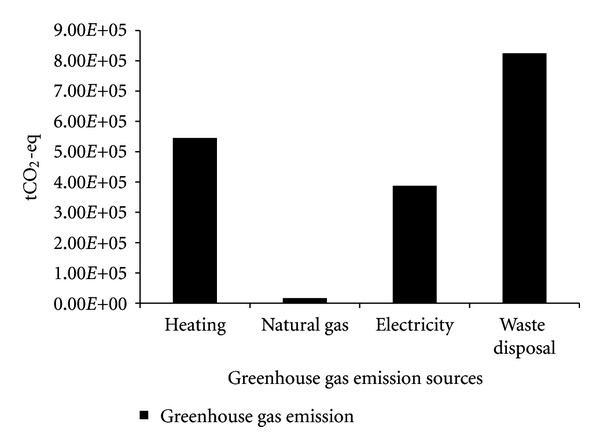
Greenhouse gas emission sources of the operation phase.

Based on steps of the life cycle analysis introduced in Section 3, we calculated the greenhouse gas emission of the LCHE industrial park by multiplying the raw data provided by the developer and the greenhouse gas emission intensity data derived from LCB and LCE. The total GHG emission of the construction, operation, and dismantling stages is 1.79 × 10^6^ tCO_2_-equivalent. Figures 3 and 4 demonstrate the GHG emission in the construction and operation phases of the lifetime of the LCHE industrial park. The greenhouse gas emissions from the construction phase comprise a relatively small proportion compared with the operation stage. In the construction stage, the largest three emitters are the building works (59.71%), interior decoration project (20.33%), and external decoration project (11.40%), followed by the greening project (3.74%), ventilation and air conditioning system (1.78%), and road (1.09%). The other 7 projects only occupy 1.96% of the total construction emission.

The operation stage is the largest contributor of this industrial park. In the operation stage, greenhouse gas emission from waste water treatment makes up the largest proportion of total emission. Greenhouse gas emission from direct energy consumption (natural gas) only occupies a small fraction of 0.11%. Although the consumption of electricity and heat do not generate on-site greenhouse gas emission, the greenhouse gas emission embodied in the electricity and heat generation process should not be ignored. When the indirect greenhouse gas emission of electricity and heat generation is traced and included, the utilization of heat and electricity make up proportions of 31% and 22% of the total operation GHG emission. Dismantling makes up the smallest proportion at 0.56%, which is mainly caused by the consumption of diesel.

The greenhouse gas emission density of the LCHE industrial park in the construction stage is 272 kgCO_2_ eq/m^2^, indicating that 272 kg greenhouse gas emission is generated per m^2^ construction area. Compared with some traditional buildings such as new-build dwellings (403 kgCO_2_/m^2^) [35], and a 3 bedroom semi-detached house (405 kgCO_2_/m^2^) [36], the greenhouse gas emission density is much lower, representing that the LCHE industrial park has great carbon reduction potential.

The greening rate of the LCHE industrial park is 41%, which is much higher than the value set in the “Evaluation Standard for Green Building” of China (30%) [37]. As green space performs as a carbon sink, the coverage of green land is beneficial for greenhouse gas mitigation for the LCHE industrial park. Using the accounting framework of IPCC [38], the annual greenhouse gas emission absorbed by green land in this industrial park is calculated to be 106.73 tCO_2_/year.

Greenhouse gas emission intensity in the operation stage is 2.77 × 10^−3^ kgCO_2_ eq/$, which is lower than that of the construction industry (4.87 × 10^−3^ kgCO_2_ eq/$) and the Wholesale, Retail Trade and Hotel, Restaurants industry (3.36 × 10^−3^ kgCO_2_ eq/$) [39, 40]. Obviously, when one unit of economic output is delivered, less greenhouse gas is emitted from the LCHE industrial park. Thus, it can be regarded a success practice in promoting the development of a low carbon economy.

When taking dismantling into consideration, the GHG emission should exclude the emission avoided by material recycling. As the LCHE industrial park is under operation, no detailed data on the disposal of building materials are available. A principle used by Kabir et al. [41] in material disposal and recycling has been employed for the calculation of greenhouse removal rate, based on which the greenhouse gas removal rate is calculated to be 15.73%.

### 4.2. Economic Investment

**Figure 5:**
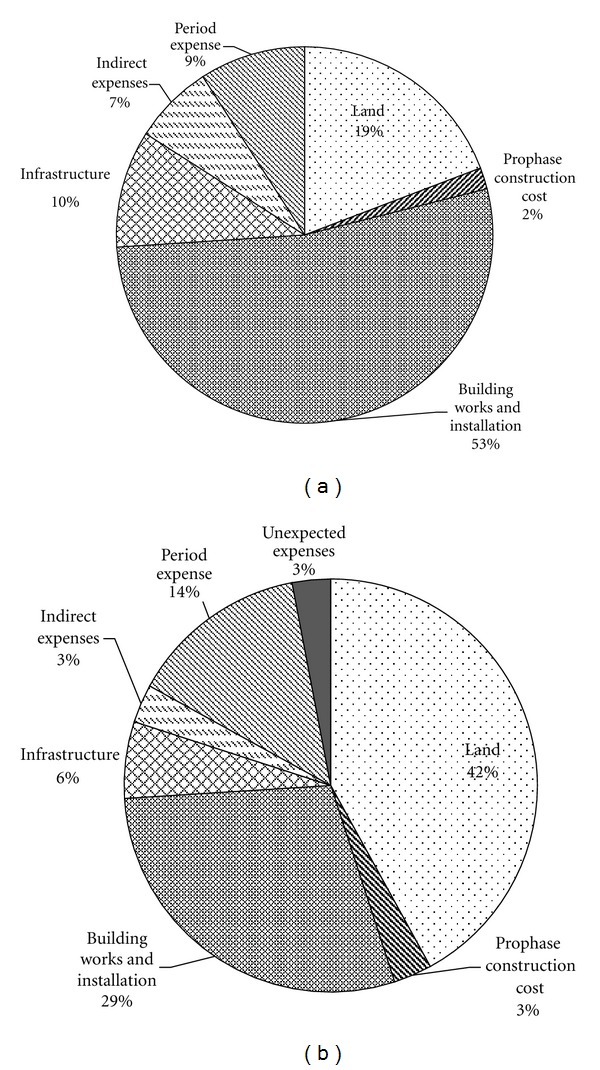
Investment structure of the LCHE industrial park and the average real estate investment in China ((a) LCHE industrial park, (b) the average China).

The total investment on the construction of the LCHE industrial park is $6.15 × 10^7^, in which the costs of prophase construction, building works and installation costs, infrastructure costs, indirect expenses, period expenses, and land fees constitute 2%, 53%, 10%, 7%, 9%, and 19%, respectively, (Figure 5(a)). Obviously, investments in building works and installation make up the largest proportion. It can be thus concluded that the fluctuations of construction material price exert the largest influence on economic indicators.

Compared with the investment structure of the average real estate in China (Figure 5(b)), the proportion of investment in land in the LCHE industrial park is relatively low. The building works and installation expenses of 53%, which is much higher than that of the average China, can be attributed to the utility of low carbon building materials, which is more expensive than traditional building materials. As a complete infrastructural facility has been installed in this LCHE industrial park, the proportion of infrastructure costs is higher than that of the average China. The indirect fee of the LCHE industrial park is lower than that of the average China, implying the necessity of improving managerial efficiency of the construction and avoiding unnecessary expenses.

**Figure 6:**
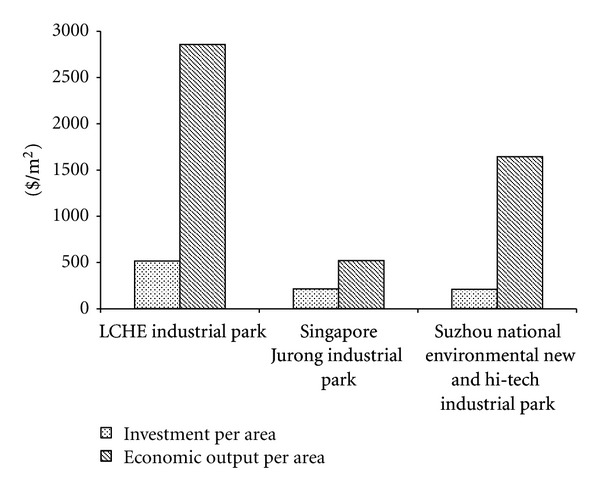
Economic indicators of different industrial parks.

Figure 6 demonstrates the economic indicators of different industrial parks. The USA dollar (exchange rate $1 = 6.3 yuan RMB) was used as the currency unit for global comparison purposes. Apparently, the LCHE has the highest investment per area, which implies that the construction of low carbon industrial park is not that economically competitive compared with other eco-industrial parks. However, the economic output per area of the LCHE industrial park is much higher than the other two eco-industrial parks. It accounts for the settlement of high-end and high valued added enterprises, which is regarded an “engine” for regional economy economic structure upgrading. High-end and high valued added enterprises are now emerging economic entities which should be emphasized in the entrance permission system of industrial parks.

### 4.3. Resource Depletion

The structure of embodied energy consumption in the construction phase is similar with the distribution of the greenhouse gas emission discussed in Section 4.1. In the operation phase, the heat consumption makes up the largest proportion, followed by the electricity consumption. Energy consumption of natural gas and the waste disposal only constitute a small fraction. In the dismantling phase, the embodied energy consumption constitutes only 0.71%, which can be neglected.

Energy density of the LCHE industrial park is calculated to be 1.31 GJ/m^2^, much lower than the conventional dwelling building (3.25 GJ/m^2^) [42], which implies that the LCHE industrial park is a promising approach in reducing energy depletion in the building industry. The energy intensity in the operation stage is calculated to be 0.12 GJ/m^2^. The comparisons of heat and electricity consumption with other EIPs are listed in Table 2. The heat density of the concerned LCHE industrial park is higher than some manufacturing and tertiary industries, and lower than the transportation industry. Heat consumption in this industrial park should thus be controlled. The electricity consumption density of the LCHE industrial park is the lowest, meaning that the electricity consumption in the LCHE industrial park can satisfactorily meet the demand of low carbon and energy-saving lifestyle.

In the LCHE industrial park, waste water generated in the operation phase is treated by the waste water treatment system. The treated water is then recycled and reused to satisfy daily water demand in the park. In the “Evaluation Standard for Green Building” of China, the proportion of unconventional water should constitute more than 10% of the total water consumption [37]. The water recycling rate of the LCHE industrial park is 50.80%, which is much higher than the national standard of green buildings.

Owing to material recycling, energy embodied in materials like steel, copper, and so forth, can be recycled and reused. According to the discipline of Kabir et al. [41], energy reduction rate of the LCHE industrial park is 10.30%, which is lower than the specified 30% for green buildings [37]. Further plans that can improve the energy reduction rate should be made to qualify a low carbon and energy-saving industrial park in terms of dismantling and disposal.

## 5. Conclusions

Along with the enforcement of the blossom of low carbon economy in China, the eco-industrial parks and low carbon industrial parks have been developing in an unprecedented way. To probe into the sustainability of low carbon industrial parks and make tradeoffs with traditional parks, in this paper, embodied energy, greenhouse gas emission, and economic aspects of sustainability for a LCHE industrial park in Beijing, China, are monitored by proposing an evaluation framework with a series of indicators. In combination with the life cycle analysis, the evaluation framework assessed the whole lifetime sustainability of the industrial park.

From the embodied energy use and greenhouse gas emission perspectives, compared with other industrial parks or green buildings, it is obvious that the LCHE industrial park is a good choice for both energy savings and greenhouse gas emission reduction. In addition, in the whole lifetime of the LCHE industrial park, the greenhouse gas emitting from operation stage contributes most to the total emissions and embodied energy consumption. This indicates that the emphasis of low carbon industrial park management should be laid on building up a low-carbon lifestyle.

In addition, in the construction phase, building works is the main component that affects the environment and resource use most. Thus, the selection of low carbon material is the key point. Meanwhile, manufacturers should pay attention to the optimization of manufacturing processes in order to produce low carbon products. It is also promising to look more closely into ways of material recycling and reusing building materials, which may make great contributions to both energy saving and greenhouse gas emission reduction.

Based on the economic indicators, the overall results indicate that the investment is relatively higher than that of other industrial parks. There is thus a necessity of improving managerial efficiency of the construction and avoiding unnecessary expenses. In view of profit gained, the LCHE industrial park is much more economically profitable owing to the settlement of high end enterprises. This is a new economic cluster mode which will be an inevitably prevalent trend in China.

Moreover, this indicator system is only a preliminary work, which should be further improved, for example, to make the social aspect of sustainable development included. Also, more case studies should be conducted and compared to make tradeoffs and shed light on future development pathways of industrial parks.

## Figures and Tables

**Table 1:** Sustainability evaluation indicators in the multicriteria scheme.

Categories	Stages	Indicators	Units	Explanation
		Greenhouse gas emission density	kg CO_2_ eq/m^2^	The greenhouse gas emission in the construction phase divided by construction area.
Environmental	Construction	The greening rate	%	The proportion of green land to the total construction area of industrial parks.
	Operation	Greenhouse gas emission intensity	kg CO_2_ eq/$	The greenhouse gas emission in the operation phase divided by economic output.
	Dismantling	Greenhouse gas emission removal rate	%	The greenhouse gas emission avoided by material recycling divided by total greenhouse gas emission in the construction and operation stages.

	Construction	Energy density	J/m^2^	Energy consumption per construction area in the construction phase.
Resource		Water recycling rate	%	The proportion of reused water to total water consumption.
	Operation	Energy intensity	J/$	Energy consumption per economic output in the operation phase.
	Dismantling	Energy reduction rate	%	Energy recycled in the dismantling phase divided by total embodied energy consumption.

Economic	Construction	Investment per area	$/m^2^	All expenses that support the construction of the industrial parks per built-up area.
	Operation	Economic output per area	$/m^2^	Economic output produced by settled enterprises per m^2^.

**Table 2:** Comparisons of heat and electricity consumption of different industrial parks [32].

Industrial parks	Heat consumption density per year (GJ/m^2^)	Electricity consumption density per year (GJ/m^2^)
The LCHE industrial park	1.08	0.15
Wood import and manufacturing of wooden playgrounds	0.72	0.36
Manufacturing of moulds	0.72	0.36
Retail and distribution of sports equipment	0.54	0.5
Transportation and storage	1.45	0.69
Distribution of agricultural products	1.71	4.66
